# Performance comparison of phenotypic and molecular methods for detection and differentiation of *Candida albicans* and *Candida dubliniensis*

**DOI:** 10.1186/1471-2334-12-230

**Published:** 2012-09-25

**Authors:** Suhail Ahmad, Ziauddin Khan, Mohammad Asadzadeh, Ajmal Theyyathel, Rachel Chandy

**Affiliations:** 1 Department of Microbiology, Faculty of Medicine, Kuwait University, P. O. Box 24923, Safat, 13110, Kuwait

**Keywords:** *Candida albicans*, *Candida dubliniensis*, Detection, Differentiation, Duplex PCR

## Abstract

**Background:**

*Candida albicans* is the most pathogenic *Candida* species but shares many phenotypic features with *Candida dubliniensis* and may, therefore, be misidentified in clinical microbiology laboratories. Candidemia cases due to *C. dubliniensis* are increasingly being reported in recent years. Accurate identification is warranted since mortality rates are highest for *C. albicans* infections, however, *C. dubliniensis* has the propensity to develop resistance against azoles more easily. We developed a duplex PCR assay for rapid detection and differentiation of *C. albicans* from *C. dubliniensis* for resource-poor settings equipped with basic PCR technology and compared its performance with three phenotypic methods.

**Methods:**

Duplex PCR was performed on 122 germ tube positive and 12 germ tube negative isolates of *Candida* species previously identified by assimilation profiles on Vitek 2 ID-YST system. Typical morphologic characteristics on simplified sunflower seed agar (SSA), and reaction with a commercial (Bichro-Dubli) latex agglutination test were also performed. The assay was further applied on 239 clinical yeast and yeast-like fungi and results were confirmed by DNA sequencing of internal transcribed spacer (ITS) region of rDNA.

**Results:**

The results of duplex PCR assay for 122 germ tube positive and 12 germ tube negative isolates of *Candida* species were comparable to their identification by Vitek 2 ID-YST system, colony characteristics on SSA and latex agglutination test. Application of duplex PCR also correctly identified all 148 *C. albicans* and 50 *C. dubliniensis* strains among 239 yeast-like fungi.

**Conclusions:**

The data show that both, duplex PCR and Bichro-Dubli are reliable tests for rapid (within few hours) identification of clinical yeast isolates as *C. dubliniensis* or *C. albicans*. However, duplex PCR may be applied directly on clinical yeast isolates for their identification as *C. dubliniensis* or *C. albicans* as it does not require prior testing for germ tube formation or latex Candida agglutination.

## Background

*Candida albicans* is the predominant pathogenic *Candida* species in susceptible human host. Infections caused by non-*albicans Candida* spp. have, however, also increased in recent years and may account for up to 60% of all episodes of candidemia or invasive candidiasis at some centers [1,2]. *Candida dubliniensis*, first described in 1995 from oral cavities of human immunodeficiency virus-infected individuals [3], is increasingly being reported from patients with candidemia in recent years [1,2,4]. *C. dubliniensis* shares several phenotypic characteristics, such as ability to form chlamydospores on cornmeal agar and germ tubes in serum with *C. albicans*. Since germ tube test is routinely used for the differentiation of *C. albicans* from other *Candida* species, identification based solely on this test leads to misidentification of some *C. albicans* isolates in routine diagnostic laboratories [5]. Accurate identification is warranted since *C. dubliniensis* exhibits increased adherence to buccal epithelial cells and is more likely to develop resistance against fluconazole and other azoles [6,7].

Several phenotypic tests based on colony morphology and physiological assimilation tests have been developed to distinguish *C. albicans* from *C. dubliniensis.* Commercially available yeast identification systems (Vitek 2 ID-YST, API 20C and ID32C) based on utilization of various compounds have been used for differentiating various *Candida* spp., however, these methods are expensive and require at least 1–2 days to report results [8]. Formation of rough/fringed colonies by germ tube positive isolates on differential media such as Niger seed agar [9], simplified sunflower seed agar (SSA) [10], or tobacco agar [11], or inability to grow in a hypertonic Sabouraud dextrose broth containing 6.5 M NaCl [12] are often used to differentiate *C. albicans* from *C. dubliniensis*. A commercial latex agglutination test (Bichro-Dubli Fumouze) is also available [13]. However, prior presumptive identification of *C. albicans/C. dubliniensis* is required before phenotypic methods could be used for their differentiation. Also, variations in growth conditions (incubation temperature, repeated subculturing and storage) may impede accurate identification of these two species by phenotypic tests. Unambiguous identification can be established only by molecular techniques and PCR-based methods are mostly employed [5]. Matrix-assisted laser desorption ionization-time of flight mass spectrometry (MALDI-TOF MS) has also been exploited recently for identification and differentiation of cultured yeast isolates including even closely related *Candida* spp. [14]. However, the high cost of the equipment is a major impediment for its widespread use in resource-limited settings. In this study, we have developed a novel duplex PCR assay by using primers derived from unique rDNA sequences for rapid detection and differentiation of *C. albicans* and *C. dubliniensis*. The performance of duplex PCR was compared with three phenotypic methods for identification of clinical isolates as *C. albicans* or *C. dubliniensis*.

## Methods

### Reference strains and clinical isolates

*Candida dubliniensis* (CD 36/CBS 7987/ATCC MYA-646), *C. albicans* (ATCC 90028), *Candida parapsilosis* (ATCC 10233), *Candida glabrata* (ATCC 15545), *Candida tropicalis* (ATCC 750) and *Candida krusei* (CBS 6258) were used as reference *Candida* species. One hundred thirty-four clinical isolates representing *C. dubliniensis* (n = 67), *C. albicans* (n = 55), *C. parapsilosis* (n = 2), *C. glabrata* (n = 2), C. *tropicalis* (n = 2), *Candida lusitaniae* (n = 2), *Candida krusei* (n = 2) and *Candida kefyr* (n = 2) were tested by both phenotypic and molecular tests. All isolates of *C. albicans* and *C. dubliniensis* were positive by germ tube test. Identity of *C. dubliniensis* strains was also confirmed by sequencing of internal transcribed spacer (ITS) region (containing ITS-1, 5.8S rRNA and ITS-2) of rDNA as described previously [15]. Additionally, 239 clinical yeast isolates including *C. albicans* (n = 148), *C. dubliniensis* (n = 50), *C. parapsilosis* (n = 5), *C. glabrata* (n = 5), *C. lusitaniae* (n = 5), *C. tropicalis* (n = 5), *Candida haemulonii* (n = 3), *Candida rugosa* (n = 2), *Trichosporon* species (n = 4) and *Cryptococcus* species (n = 6) and one isolate each of *C. krusei*, *Candida guilliermondii*, *C. orthopsilosis*, *C. metapsilosis*, *C. nivariensis* and *C. bracarensis* were also tested by duplex PCR. The isolates were cultured as part of standard patient care and no individual patient approval was needed for their subsequent use. The project under which the study was performed was approved by the Ethical Committee of Faculty of Medicine, Health Sciences center, Kuwait University.

### Phenotypic identification and characterization

Clinical isolates were speciated by carbohydrate assimilation profile using Vitek 2 ID-YST system (bioMerieux, Marcy-lEtoile, France) and tested for germ tube formation in horse serum. All germ tube positive isolates were presumptively identified as *C. albicans*/*C. dubliniensis*. Germ tube positive isolates showing fringed/rough colonies on SSA [10] were identified as *C. dubliniensis*. Bichro-Dubli (Fumouze Diagnotics, Levallois-Perret, France) latex agglutination test for identification of *C. dubliniensis* strains was performed on all germ tube positive isolates according to manufacturer’s recommendations.

### Template DNA preparation, duplex PCR assay and DNA sequencing

For molecular studies, a loop full of yeast colony from Sabouraud dextrose agar plate was suspended in 1 ml of sterile water in a microcentrifuge tube containing 50 mg Chelex-100 (Sigma-Aldrich Co.), the contents were heated at 95°C for 20 min and then centrifuged. The supernatant was transferred to a new tube and typically 2 μl was used in PCR. Species-specific identity of *C. albicans* and *C. dubliniensis* strains was performed by duplex PCR by using primers targeting sequences in ITS-1 and ITS-2 regions of rDNA. Species-specificity of primer pairs CALF (5′-TGGTAAGGCGGGATCGCTT-3′) + CALR (5′-GGTCAAAGTTTGAAGATATAC) and CDUF (5′-AAACTTGTCACGAGATTATTTTT) + CDUR (5′-AAAGTTTGAAGAATAAAATGGC-3′) for *C. albicans* and *C. dubliniensis*, respectively, was indicated by BLAST searches ( http://www.ncbi.nlm.nih.gov/BLAST/Blast.cgi?). PCR amplification was performed in a final volume of 50 μl containing 1x AmpliTaq DNA polymerase buffer I and 2 units of AmpliTaq DNA polymerase (Perkin Elmer), 10 pmol of CALF + CALR + CDUF + CDUR primers, 2 μl of template DNA and 100 μM of each dNTP. Cycling conditions included an initial denaturation at 95°C for 5 min followed by 30 cycles of 95°C for 1 min, 55°C for 30 s and 72°C for 1 min and a final extension at 72°C for 10 min. PCR products (20 μl) were run on 2% (w/v) agarose gels, as described previously [16]. The species-specific identity of clinical yeast isolates was established by sequencing of ITS region of rDNA. The ITS region was amplified by using panfungal primers ITS1 [17] and CTSR [18] and the amplicons were purified and sequenced as described in detail previously [15,19].

## Results

**Figure 1:**
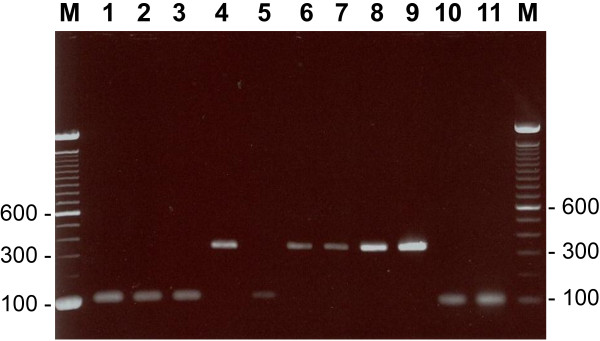
**Agarose gel of duplex PCR assay from representative isolates of *C. albicans *(lanes 1–3, 5, 10, 11) and *C. dubliniensis *(lanes 4, 6–9).** No amplification was obtained in control (no DNA added) tubes. The duplex PCR test was uniformly negative for other *Candida* species or yeast-like fungi. Lane M is 100 bp DNA ladder and the positions of migration of 100 bp, 300 bp and 600 bp fragments are marked.

Duplex PCR amplification with CALF + CALR + CDUF + CDUR primers yielded an amplicon of ~100 bp and ~325 bp only with DNA from reference strains of *C. albicans* and *C. dubliniensis*, respectively, but not from other *Candida* species, as expected (data not shown). Of 134 clinical *Candida* spp. isolates, 122 were positive for germ tube formation and 12 were germ tube negative. Vitek 2 ID-YST system identified the isolates as *C. dubliniensis* (n = 67), *C. albicans* (n = 55), *C. parapsilosis* (n = 2), *C. glabrata* (n = 2), C. *tropicalis* (n = 2), *C. lusitaniae* (n = 2), *C. krusei* (n = 2) and *C. kefyr* (n = 2). Duplex PCR correctly identified all 67 *C. dubliniensis* and 55 *C. albicans* isolates (data from 11 selected isolates are shown in Figure 1) while negative results were obtained for the remaining 12 germ tube negative isolates (Table 1). Growth on SSA correctly identified 67 of 122 germ tube positive isolates as *C. dubliniensis*. Bichro-Dubli test was positive for 64 of 67, weakly positive for 2 of 67 and negative for 1 of 67 isolates identified as *C. dubliniensis* by Vitek 2 ID-YST system and ITS region sequencing. Bichro-Dubli test was negative for 55 of 122 germ tube positive isolates identified as *C. albicans* by Vitek 2 ID-YST system as well as 12 germ tube negative isolates of six other *Candida* species (Table 1). Thus, duplex PCR accurately identified all 55 *C. albicans* and 67 *C. dubliniensis* strains among a panel of 134 well-characterized *Candida* spp. isolates with 100% sensitivity and specificity.

**Table 1:** **Performance comparison of duplex PCR with Vitek 2 ID-YST system, Bichro-Dubli Fumouze latex agglutination test and colony characteristics on simplified sunflower seed agar (SSA) for identification and differentiation of *C. albicans *and *C. dubliniensis***

***Candida species***	**No. of isolates tested**	**No. of isolates yielding correct identification with**			
		**Vitek 2 ID-YST**	SSA*	**Bichro-Dubli**	**Duplex PCR**
*C. albicans*	55	55	0	0	55
*C. dubliniensis*	67	67	67	66	67
*C. glabrata*	2	2	Not done	0	0
*C. parapsilosis*	2	2	Not done	0	0
*C. tropicalis*	2	2	Not done	0	0
*C. lusitaniae*	2	2	Not done	0	0
*C. krusei*	2	2	Not done	0	0
*C. kefyr*	2	2	Not done	0	0

*Fringed/rough colonies during growth on simplified sunflower seed agar (SSA).

Direct application of duplex PCR on a panel of 239 clinical isolates of yeast-like fungi correctly identified all 148 *C. albicans* and 50 *C. dublininsis* strains. The remaining isolates representing other *Candida* spp. or other yeast-like fungi yielded negative results. The results were confirmed by direct DNA sequencing of ITS region of rDNA.

## Discussion

The results of duplex PCR assay described here were completely concordant with species-specific identification of *C. dubliniensis* and *C. albicans* strains by Vitek 2 ID-YST system and colony characteristics (fringed/rough colonies) on SSA. However, both, Vitek 2 ID-YST system and growth on SSA usually require 24–48 hours before results are available. Furthermore, identification of *C. dubliniensis* strains on SSA requires prior testing for germ tube formation while Vitek 2 ID-YST system is expensive and thus, is not readily available in resource-poor settings [10]. The requirement for minimal amount of genomic DNA for duplex PCR allows the whole procedure to be completed within 4 hours. Furthermore, since duplex PCR employs species-specific primers for both, *C. albicans* and *C. dubliniensis,* it can be directly applied on clinical isolates of yeast and yeast-like fungi. Indeed, direct application of duplex PCR on a panel of 239 clinical isolates of yeast-like fungi correctly identified all 50 *C. dublininsis* and 148 *C. albicans* strains.

Although several PCR-based methods using rDNA as target have been described previously, they either involve two separate PCR reactions for each strain [16,20,21] or further manipulations (such as restriction digestion to generate restriction fragment length polymorphism or DNA sequencing) are needed for species-specific identification [16,22-24]. These additional steps add to the cost of the test and/or consume additional time thus delaying results. Other investigators have used PCR-based methods targeting intronic sequences for differentiation of *C. albicans* from *C. dubliniensis*[25-27]. However, these approaches may lead to misidentification of some isolates since intronic sequences may vary significantly [27]. A real-time PCR assay using melting point analysis was described recently for detection and differentiation of *C. albicans* and *C. dubliniensis* strains but involves separate reactions for each *Candida* spp. and expensive probe primers [28]. Although two real-time PCR assays using SYBR Green dye have also been described, these assays either require prior presumptive identification of *C. albicans*/*C. dubliniensis* strains by another (such as germ tube) test and/or two rounds of real-time PCR assays are needed [29,30]. Furthermore, the difference in *T*_*m*_ values for *C. albicans* and *C. dubliniensis* strains in the two reports was either small (<0.5°C) or negligible which may lead to misidentification of some isolates.

Performance of another rapid (Bichro-Dubli Fumouze latex agglutination) test was also compared with duplex PCR results. The 98.5% sensitivity and specificity of 100% of Bichro-Dubli test is in agreement with the reported sensitivity (97% to 100%) and specificity (100%) values suggested by the kit manufacturer. Similar values have also been reported in other studies for identification of *C. dubliniensis* isolates [31,32]. Although Bichro-Dubli test is also a rapid method for identification of *C. dubliniensis*, the test can only be applied on isolates previously identified as *C. albicans/C. dubliniensis* either by germ tube formation of by application of another (Bichro Latex Albicans) latex agglutination test [13,32]. This increases both, the cost and the time to report results for Bichro-Dubli test. The use of chromogenic differential media such as CHROMagar® *Candida* has also been implemented in many clinical microbiology laboratories for identification and differentiation of both *C. albicans* and *C. dubliniensis*, thus diminishing the need for germ tube test. However, the production of light green (for *C. albicans*) or dark green (for *C. dubliniensis*) color is inconsistent when large number of isolates are screened, thus requiring further cultivation on a bird-seed agar medium for their accurate identification [33].

## Conclusions

A duplex PCR assay has been developed and extensively evaluated for the detection and differentiation of clinical isolates as *C. dubliniensis* or *C. albicans*. The method may be applied directly on clinical yeast isolates for their identification as *C. dubliniensis* or *C. albicans* as it does not require prior testing for germ tube formation or other such tests and is suitable for resource-limited clinical microbiology laboratories equipped with basic PCR technology. A limitation of the study is that other *Candida* and yeast-like organisms will require additional testing for their species-specific identification.

## Abbreviations

ITS: Internal transcribed spacer; SSA: Simplified sunflower seed agar; rDNA: Ribosomal DNA.

## Competing interests

The authors declare that no competing interests exist.

## Authors’ contributions

SA and ZK designed the study, arranged financial support and analyzed the data. MA, AT and RC performed the experiments. All authors contributed in writing the manuscript and approved the final version.

## Pre-publication history

The pre-publication history for this paper can be accessed here:

http://www.biomedcentral.com/1471-2334/12/230/prepub
